# Health SDGs are at risk from climate change: Evidence from India

**DOI:** 10.1371/journal.pone.0335529

**Published:** 2025-11-26

**Authors:** Purnamita Dasgupta, Girika Sharma, William Joe, Madhura Chowdhuri, Gudakesh G

**Affiliations:** Institute of Economic Growth, Delhi, India; Jawaharlal Nehru University, INDIA

## Abstract

Climate change poses significant risks to human health in India, with 80% of the population located in areas highly vulnerable to extreme events, such as cyclones, floods and heatwaves. While India has made progress on several Sustainable Development Goals (SDGs), risks from climate change can undermine the achievements. This study examines the impact of climate vulnerability on health related targets under the SDG2 on Zero Hunger and SDG3 on Good Health and Well-being. Statistical and econometric methods including a multivariate logistic regression are used to examine the relationship between climate vulnerability, social and economic determinants of health and health outcomes in 575 districts of rural India. 2 national datasets are used for the analysis, namely, a health survey and a climate risk and vulnerability assessment, with a sample size of 154,547 children and 447,348 women. A highly significant and negative relationship is found between climate vulnerability and attainment levels of health outcomes. Districts that are highly vulnerable to climate change consistently underperform on the studied health targets as compared to districts which are less vulnerable to climate change. For instance, the chance of children being underweight and that of women having non-institutional deliveries, is 1.25 and 1.38 times higher, respectively, in districts that are highly vulnerable to climate change than districts that are less vulnerable. While the extent of the adverse impact varies, the findings establish the necessity to take account of the adverse impacts of climate change on health outcomes, apart from the socio-economic and access related factors that have conventionally been considered as relevant in influencing these outcomes. in LMICs like India. There is an urgent need for timely action to address climate change risks, including effective adaption in health, to ensure that the desired health and well-being outcomes can be achieved and sustained, amidst rising climate risks.

## 1. Introduction

Risks to human health from climate change are on the rise globally, adversely impacting and undermining multiple dimensions of health and well-being [1,2], including in India [3,4]. Climate change impacts on health include increasing mortality and morbidity from extreme events such as heatwaves [2,5], droughts, and floods [2]; spread of vector borne diseases [6,7] and altered disease patterns, disruptions in access to health care [8], destruction of health infrastructure [8]; and impacts on health-seeking behavior [9,10], to name a few. For instance, evidence shows that long term exposure to extreme heat increases the risk of death from existing illnesses including cardiovascular and respiratory illnesses, along with the direct impacts, such as exhaustion, headache, sleep disturbances and heat stroke [11–13].

Climate risks can adversely impact the achievement of the Sustainable Development Goals (SDGs) of the UN [14]. The SDGs comprise of seventeen global goals and targets enshrined under each goal, and are intended to ensure health, justice and prosperity [15]. Health is an important component of the SDGs, with SDG 2 (Zero Hunger) and SDG 3 (Good Health & Wellbeing) containing several health-related targets, such as ending hunger, improving nutrition, reducing mortality, improving access to healthcare, and ensuring universal health coverage. It is critical to accelerate progress in achieving the health targets of the SDGs, since the current trends in these are insufficient to meet the health goal by 2030 [16]. Climate change, climate-related risks, and disasters can pose significant challenges to achieving the SDG targets [17], and potentially disrupt the achievement of SDGs [18,19].

For effectively addressing shortfalls and reducing disparities in health amongst vulnerable populations, it is therefore essential to take serious note of these impacts and drive investments in the right direction. The extent of vulnerability differs from one region to another, especially in a large country like India, and is influenced by local climatic, geographical, and socio-economic factors [20]. Around 64% [21] of the Indian population stays in rural areas while 46% of the population is employed in agriculture in 2023 [22]. As compared to urban areas, rural areas are under-served in terms of health care services [23] and infrastructure such as roads, electricity, housing and communications [24]. The gap in consumption expenditure between rural and urban areas has been reducing [22] Given the significant share of population residing in rural areas, estimated to be around 915 million in 2023 [25] mapping and quantifying the shortfalls in health outcomes which are attributable to climate change impacts can help develop appropriate adaptation policies. The impacts of climate hazards on human health are severe and already being felt globally as well as in India [26–29]. This study is an innovative attempt merging data from the largest national survey on health with climate data, to examine and provide quantifiable evidence on the links between climate and health outcomes in India. It examines whether climate action can contribute to achieving good health.

The objective of the study is thus set-up to provide quantitative evidence on the extent to which climate change impacts and poses a barrier in achieving the health-related SDG targets across different districts in rural India. The data used for the analysis is taken from national level surveys conducted by government agencies and is available in the public domain. Standard statistical and econometric techniques are used for the analysis. The health-related targets have been selected from those enshrined under the SDG 2 on Zero Hunger and SDG 3 on Good Health and Wellbeing. The specific targets which were studied include reduction in malnutrition in terms of stunting, wasting, and being underweight for kids under the age of 5 from SDG 2, and, decrease in the share of non-institutional deliveries and problems in accessing healthcare for women under SDG 3. The impact of climate change is taken from district level climate vulnerability data while health and socio-economic data at the district level are available from a demographic survey. By examining the relationship between climate vulnerability, socio-economic indicators and health indicators, the study seeks to shed light on how vulnerability to climate change impacts efforts to reduce disparities and promote well-being in health outcomes.

### Relevant findings from a desk review

The literature on non-climatic determinants influencing the attainment of health-related targets is well-established. We present below some of the key findings from the literature on these key determinants.

Malnutrition alone is responsible for 45 percent of deaths among children under the age of five [30] and continues to be a significant challenge in many parts of the world, with an estimated 149 million children under the age of five stunted, 45 million wasted, and 37 million overweight or obese as of 2022 [30]. It is concentrated in low- and middle-income countries, particularly in sub-Saharan Africa and South Asia. For India, the SDG 2 index score is 52, much below the target score of 100 [31]. The prevalence of stunting, being underweight, and chronic malnutrition in India is high, with significant regional disparities [32,33]. Around 36% of children under the age of five years were stunted, 32% were underweight, and 19% were wasted in India as per the National Family Health Survey 2019–2021 [34]. Poverty, poor sanitation, lack of access to healthcare, and inadequate education are among the key factors that contribute to the high burden of malnutrition in India, and elsewhere [35]. Malnutrition indicators also vary by demographic factors such as gender, age, education of the mother and the birth order of the child.

The understanding on the socio-economic and cultural determinants of malnutrition has advanced significantly. Studies, both globally and in India, have found that female children (under the age of 5 years) are relatively more vulnerable to malnutrition than males due to various socio-economic and cultural factors that lead to prioritization of the needs of male children [36,37] Lower levels of parental education, particularly maternal education is also significantly associated with higher malnutrition rates among children [38] with education providing mothers with better knowledge of nutrition, health practices, access to healthcare [39,40] and implementation and utilisation of these for enhancing nutritional outcomes for children [41]. In India and elsewhere, maternal education, employment and socioeconomic status interact in many ways [42,43], for instance, higher maternal education can reduce the negative effects of low household wealth on child nutrition [44]. A study in Uttar Pradesh, India, found that the prevalence of stunting was significantly lower among children whose mothers had completed at least primary education [45]. Globally, evidence indicates that stunting, wasting, and being underweight are more prevalent among male children born to mothers with no schooling, and amongst those from lower-income quintiles [46].

Studies in some African countries found that children with siblings or with a higher birth order were more likely to be stunted and underweight [47]. A few Indian studies report similar findings that a higher birth order increases the likelihood of children being stunted and underweight [48,49]. Global evidence shows that in many households, resources such as food, attention and healthcare are spread thinner with more children and in some cases, are distributed based on birth order [50,51].

Globally, studies indicate that increased antenatal care (ANC), particularly when provided by skilled healthcare professionals, correlates positively with reduced risks of malnutrition among children, especially in low and middle-income countries [52], reducing neonatal and infant mortality [53], low birth weight [52], stunting [54], and prevalence of underweight children [55].

India’s performance has been comparatively better in SDG 3, with an index score of 77 as compared to a target of 100 [31]. Significant advancements in maternal and child healthcare services have come from concerted efforts through programs and interventions under the National Health Mission [56,57], and targeted initiatives from the government focusing on high quality, comprehensive ANC while alleviating financial burdens on women [58]. Some schemes incentivize institutional childbirth and mitigate direct expenses [59], while others focus on improving the quality of care and services during labor [60]. Child mortality rates have reduced significantly in India [61,62] due to some of these efforts, though disparities persist in some regions and for specific disadvantaged groups [63].

Institutional deliveries in India have significantly increased over the past three decades with most states having already surpassed SDG targets (92%) for 2030. However, challenges persist in some regions, with institutional delivery rates below 80%. A lack of ANC, poor socioeconomic status, locational disadvantages, and higher birth order are among the challenges associated with lower utilization of institutional delivery in certain regions [64–66], manifested in terms of poor performance in key indicators [67–69]. Access to healthcare is a crucial determinant for achieving good health and health equity. Several studies in South Asia, including India, have identified access to health care as being critical for health promotion and health equity, and for addressing vulnerabilities across communities and overcoming gender disparities in health outcomes. Harsh geographic conditions, long distances to health centers, limited transportation, lack of services, absence of adequate and trained health care professionals, and financial barriers have been often noted as impacting access to equitable and quality health care [70–74]. Increasingly, climate change has become a major force disrupting health care delivery, undermining the social determinants of good health and reducing the capacity to provide health care, including universal health coverage [15], with the potential of significantly increasing health costs in LMICs [75,76].

Climate change is thus increasingly recognized globally as a major determinant of health, with differential and adverse impacts for vulnerable populations, including women and children. The need to address the impacts of climate change on health along with the other socio-economic determinants of health is being recognized [77]. In India, are preparing action plans on climate change and human health. However, the evidence base and understanding on the impacts due to climate change is weak. Prior to the current study, there have been no such attempt at quantifying the available evidence in terms of the impacts on human health in India at an All-India level, with sufficient granularity to understand differential impacts at sub-national levels.

## 2. Materials and methods

### Methods

The study uses statistical and econometric methods to examine the relationship between climate change and health outcomes. A multivariate logistic regression is estimated. Such estimation techniques have been used elsewhere to examine the relationship between climate change variables and health outcomes, such as in USA and China [78,79].The health-outcomes studied are *stunting, wasting,* and being *underweight* for children under five years of age, and *non-institutional deliveries* and *problems in accessing health care* for women aged 15–49 years. The role of climate change as a determinant for these health outcomes is captured by an explanatory variable on climatic vulnerability across districts. Other socio-economic variables are also included as determinants in the specification.

The outcome in the logistic equation is specified as a dichotomous dependent variable taking a value of 0 or 1, depending on whether the outcome is observed or not (e.g., 1 if stunted, 0 if otherwise). The model provides the probability (*p*) that the event (e.g., stunting) will occur relative to the probability of its non-occurrence (1-*p*), providing the odds of the event occurring, and the odds ratio, with a reference category [80]. In a multivariate specification, each odds ratio [81] provides an estimate of the change in the odds or chances (e.g., of a child being stunted) that is associated with the corresponding explanatory variable (*x*_*i*_, e.g., climatic vulnerability), while controlling for the other independent variables (e.g., gender, age, mother’s education).Using a logarithmic transformation of the odds ratio (e.g., odds of being stunted), a multivariate specification generates the log odds ratio for each explanatory variable in the model. Equation 1 presents the general form of the specification used. *β*_*i*_(i = 1,2….k) denotes the coefficient of the corresponding independent variable, *β*_0_ is the intercept term and *X*_i_ represents the independent variables in the model*. p* is the probability of the outcome (for instance: if the child is stunted, then *p* = 1). The term (*p/*1-*p*) denotes the odds of the outcome occurring, while log(*p*/1-*p*) represents the log-odds (logit) of the outcome.


$$\text{log}(\text{p}/\text{1}-\text{p})=\beta_{\text{0}}+\beta_{\text{1}}\;{\text{X}}_{\text{1}}+\beta_{\text{2}}\;{\text{X}}_{\text{2}}+\cdots +\beta_{\text{k}}{\text{X}}_{\text{k}}$$ (1)

The model is applied to a combined dataset, obtained by merging two datasets, namely the National Family Health Survey-Round 5 (referred to hereafter as NFHS) [82] and the Central Research Institute for Dryland Agriculture (CRIDA) [83] datasets. The former provides information on the progress in the SDG targets, the individual and household level information on health status, socio-economic variables of interest, and on access to health care. The CRIDA dataset provides information on the climate vulnerability across districts. District boundaries for the NFHS and the CRIDA datasets were matched and aligned to ensure consistency in merging the datasets. The data for stunting, wasting, being underweight and non-institutional deliveries is extracted from the Kids Recode (KR) file of the NFHS, while the data for the ‘problems with access to healthcare’ variable is extracted from the Individual Recode (IR) file for women. The IR and KR data files were merged with the climate vulnerability dataset at the district level, across 575 districts. There are 154,547 observations for children, and 447348 observations on women available in the merged datasets. The statistical and econometric estimations on the datasets were conducted using Stata software, version 16.

### Ethics statement

This manuscript and its contents are based on publicly available, fully anonymized, secondary data. No human participants were involved and informed consent requirements do not apply. This study is one component of a larger study approved by the Institutional Ethics Committee of the Institute of Economic Growth, certification dated Aug 27, 2024, [226740/Z/22/Z].

### Data

#### Climate vulnerability data.

**Fig 1:**
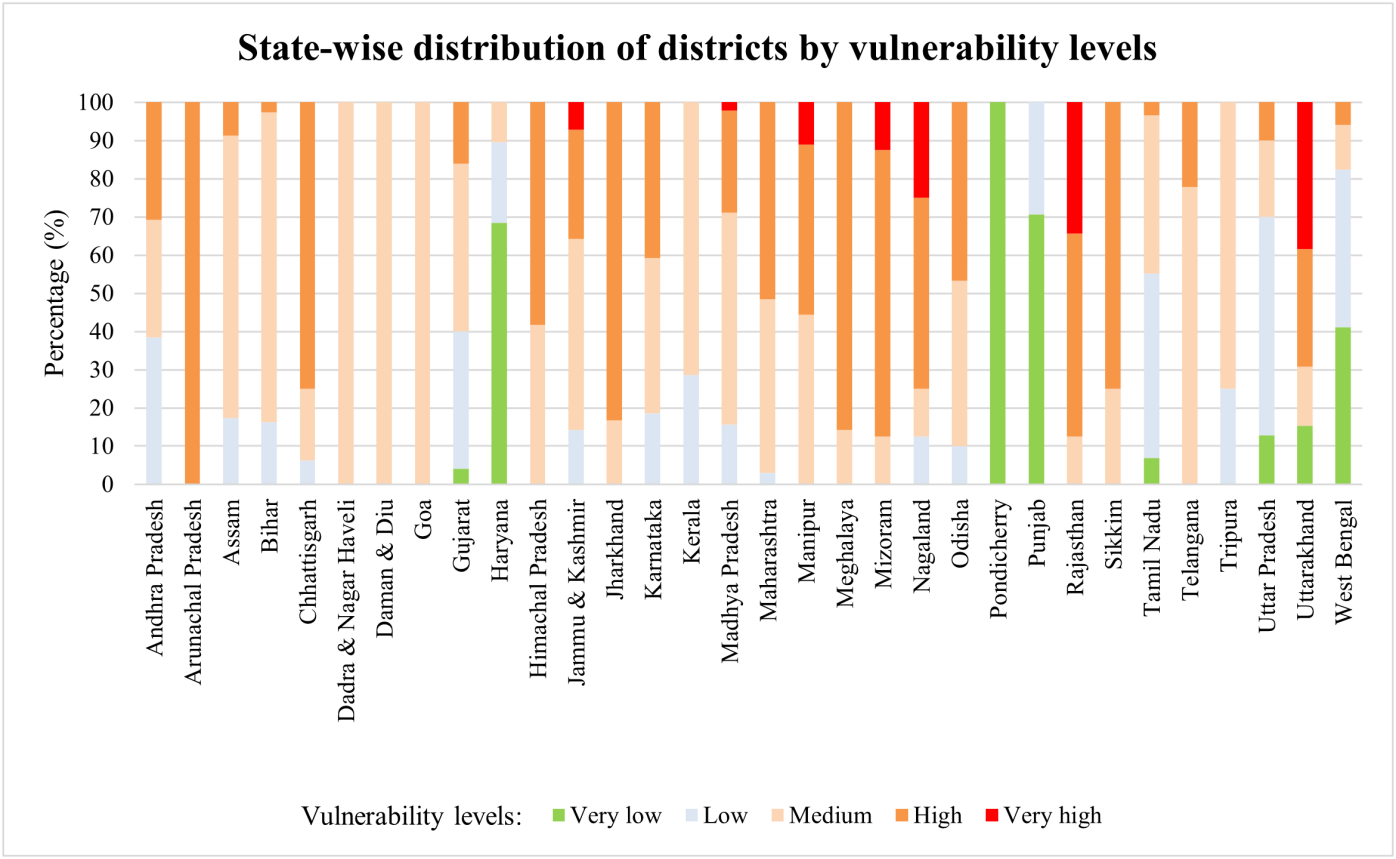
State-wise distribution of districts by vulnerability levels.

District-wise scores on climatic vulnerability were sourced from the dataset on risk and vulnerability assessment of Indian agriculture to climate change published by the. This dataset used fifteen indicators capturing five dimensions of capital endowment- natural, human, social, physical and financial, to develop a ranking on climatic vulnerability across rural districts in India. Districts are classified into five levels of vulnerability as having very high, high, medium, low or very low levels of vulnerability. Fig 1 shows the distribution of climatic vulnerability across districts in India.

Rajasthan has the highest number of districts which are either very highly or highly vulnerable, followed by Jharkhand, Chhattisgarh and Uttarakhand among the larger states. In absolute terms, the area, climatic conditions, geographic features and number of districts vary widely across states. It is revealing that the extent of vulnerability to climate change is not clustered uniquely by geographical location. Within a state, the vulnerability to climate change can vary substantially across constituent districts. For instance, among the larger states, districts in Gujarat, West Bengal, Uttar Pradesh and Uttarakhand have high within state variability in the distribution of districts by levels of climatic vulnerability. Several districts in the North Eastern region of the country have high levels of climatic vulnerability, with all districts in Arunachal Pradesh being highly vulnerable. (S1 Fig. provides the distribution of very high and highly vulnerable districts across states in India).

The percentage distribution of observations in the merged dataset is such that there are relatively very few observations falling in the very highly vulnerable districts or the very low vulnerability districts. (S1 Table 1 provides the distribution of observations by climatic vulnerability levels). It is to be noted that for the econometric estimations, the climatic vulnerability levels were re-grouped to create a binary variable with two clearly distinct categories. The very high and highly vulnerable districts were clubbed together in one category (re-named and referred to hereafter as highly vulnerable districts), and the medium, low and very low districts in another category (re-named and referred to hereafter as other districts).

#### Health data.

The health outcome variables that have been selected for the analysis are specified as binary indicators. The data is extracted from the NFHS survey, which was conducted for most states in 2019, overlapping with the year for the dataset on climate vulnerability. The Covid-19 pandemic delayed the completion of the dataset to early 2021 [84]. Any limitations arising from these circumstances are deemed to have been taken care of as the study uses the finalized and corrected dataset approved by the Government and made available in the public domain. NFHS surveys are conducted under the stewardship of the Ministry of Health and Family Welfare, Government of India. We consider only data from rural areas to maintain consistency across the NFHS and the climate datasets. The NFHS covered more than 0.6 million (636000) households, including 726,828 women aged 15–49 and 102,266 men aged 15–54. It is also to be noted that the NFHS target populations were aligned with the SDG targets, further ensuring the suitability of the dataset for the analysis in this study. This dataset is used by the Government of India for health policy planning [85] and for monitoring progress towards achieving SDG 2 and 3 [31].

The dependent variables measuring stunting, wasting, being underweight, having non-institutional deliveries and problems in accessing healthcare were directly available in the dataset. (34; the descriptions of the variables are provided in S2 Table. A variable to measure access to health care was not directly available and was therefore constructed from four individual indicators on access to health care on which data had been collected from the respondents in the survey. Respondents provided information on ‘Distance to health care’; ‘availability of health care provider’; ‘availability of drugs’; and ‘availability of female health care provider’ at the nearest government health care facility. The responses were recorded in a categorical form as a ‘Big problem; ‘Small problem’; and ‘No problem’. The variable constructed from these responses assigns a value of 1 to responses of a “big problem” and clubs together responses of a ‘Small problem’ and ‘No problem’, assigning the latter a value of 0. Subsequently, all individuals who have responded as having a “big problem” for one or more of any of the four variables, are considered to have faced a problem in accessing health care. Out of the total respondents, 46% had a big problem with the non-availability of drugs and 44% with the non-availability of health care professionals at the health centers. Distance was a problem for 29% of individuals while 35% faced a problem with non-availability of female health care providers. (S3 Table provides details on the four underlying variables).

Table 1 provides the descriptive statistics for the dependent variables used in the estimation. The percentage of children suffering from one or more forms of malnutrition ranges between 19% and 37%, constituting a fairly large share of the child population. While a relatively larger proportion of women are opting for institutional deliveries, challenges in accessing healthcare persist for approximately 50% of the respondents.

**Table 1:** Descriptive statistics of dependent variables.

Sustainable Development Goal	Target Variables	No. of Respondents in Dataset	Percentage impacted adversely	Percentage unimpacted
SDG 2: Zero Hunger	Stunting	136,959	37.22	62.78
	Underweight	139,845	32.69	67.31
	Wasting	134,255	19.02	80.98
SDG 3: Good Health and Well-being	Non-Institutional Deliveries	154,547	14.93	85.07
	Problems with Access to healthcare	447,348	59.10	40.9

Additionally, we incorporate several explanatory variables in the model, based on learnings from the literature review on determinants of health outcomes. The explanatory variables used for stunting, wasting, being underweight, and having non-institutional deliveries are the child’s age and sex, the mother’s education level, whether the mother received prenatal care from a doctor, the child’s birth order, and the number of ANC checkups. The explanatory variables for the access to health care estimation are the women’s educational level; age; marital status; availability of a person to accompany the woman to the health facility, and availability of transportation facilities. State dummies are used to account for unobserved differences among states. (S4 Table provides descriptions for the variables)

Table 2 presents the data for the explanatory variables.

**Table 2:** Descriptive data on explanatory variables from NFHS.

Explanatory Variables	Level	Percentage Distribution (%)
Explanatory Variables for Stunting, Wasting, being Underweight and Non-Institutional deliveries (number of observations = 154547)		
Age of Child	Less than 1 year (less than 12 months)	20.34
	1 years (12–23 month)	19.38
	2 years (24–35 months)	19.60
	3 years (36–47 months)	19.91
	4 years (48–59 months)	20.77
Sex of Child	Female children	48.21
	Male children	51.79
Birth Order of the child*	Birth order 1	37.16
	Birth order between 2–3	48.53
	Birth order greater than 3	14.32
Mother’s Education	Up to primary	38.22
	Higher than primary	61.78
Mother had received prenatal care from a doctor during pregnancy	Not Received	46.31
	Received	56.39
Number of antenatal visits during pregnancy	Less than four visits	46.03
	Four or more visits	53.97
Explanatory variables for ‘Problems with Access to Healthcare’ (number of observations = 447348)		
Marital Status	Not Married/others	28.05
	Married	71.95wh
Educational attainment	Up to primary	38.83
	Higher than primary	61.17
Nature of the problem in getting a person to accompany the woman to a health facility	No problem	45.85
	Small problem	34.21
	Big problem	19.94
Nature of the problem in accessing transportation for the travel to a health facility	No problem	36.75
	Small problem	35.39
	Big problem	27.87

Notes: 1. * The birth order of the child is the chronological position of the child in the birth schedule for a mother 2. The KR file (for children) is used for data related to stunting, wasting, underweight, and non-institutional deliveries, while the IR file (for individual women) was used for data related to problem in access to healthcare. Consequently, the number of observations and the descriptive statistics on variables differs for the two estimations.

Overall, the sample comprises of 447,348 women, in the age range from 15 to 49 years, with a mean age of approximately 30 years. Approximately, two-thirds of the women are educated beyond primary schooling which is a noteworthy progress made in the last decade. This is also relevant to the current analysis as education is a key determinant of child nutrition outcomes, maternal care and healthcare access. The sex ratio has also improved over time. The distribution of the children’s sample across age categories is also fairly uniform in the dataset varying between 19% and 20%. The share of children with birth orders beyond three are expectedly much lower in the sample. Majority of the women have had four or more than four visits for antenatal check-up during their pregnancy. About one-fifth of the women have problems in finding a companion to reach the health facility over one-fourth, face problems in getting transportation to reach health facilities.

## 3. Results

**Fig 2:**
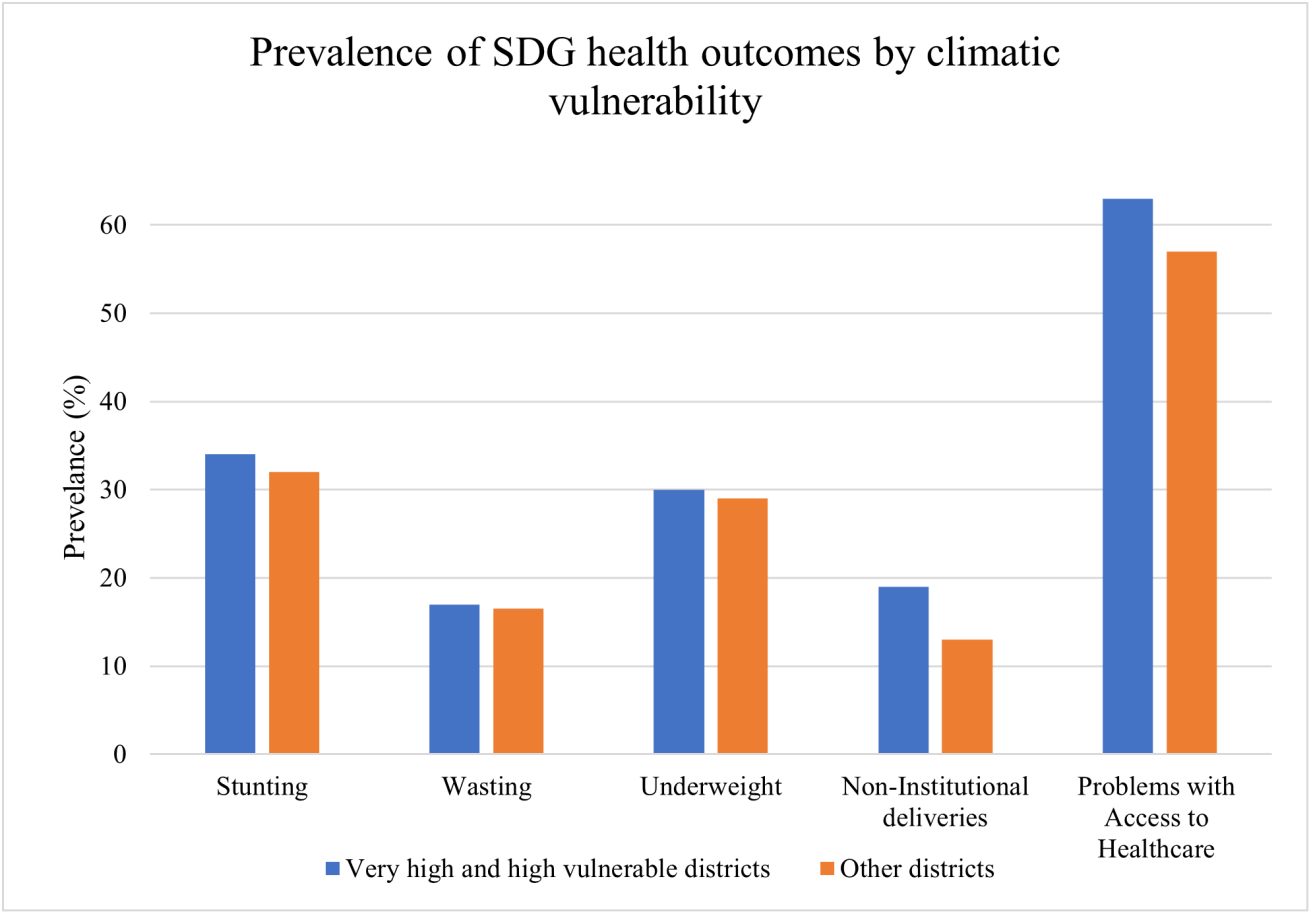
Prevalence of SDG health outcomes by climatic vulnerability.

In highly vulnerable districts, adverse health outcomes are seen to be proportionately higher in occurrence than in the other districts. Fig 2, maps the proportion of individuals with an adverse outcome in highly vulnerable districts versus those in the other districts, drawing upon the merged dataset. The blue bars represent observations from highly vulnerable districts, and the orange bars include those from the other districts. The data clearly indicates higher occurrence of adverse outcomes in districts which are highly vulnerable as compared to other districts.

The estimated log-odds coefficients corresponding to each health outcome and the standard errors from the estimated equations are presented in Tables 3 and 4. It may be noted that variance inflation factor (VIF) tests reveal that the values were less than 2, indicating very low multicollinearity among the variables (S5 Table and S6 Table provide the VIF values). Sampling weights from the NFHS were applied to all the regressions. The analysis is primarily focused on individual level determinants and avoids household level variables such as sanitation coverage and drinking water sources. There are also estimation concerns related to some of the variables that are otherwise often considered to have an influence on child health. For instance, household wealth is not used as it tends to be correlated with income, and the latter is already used in computing vulnerability. Information on variables related to dietary diversity and immunization is usually available for a sub-sample (for example, ages 6–23 months) or is heterogeneously specified (e.g., on diet) and inclusion of these would have led to reduction of the observations available for the analysis.

**Table 3:** Regression Results for – Stunting, Wasting, Underweight, and Non-institutional deliveries.

SDG Health Outcomes	(Dependent Variables)			
	Stunting	Wasting	Underweight	Non-institutional deliveries
Explanatory Variables				
Climatic Vulnerability	0.14^**^(0.02)	0.06^**^(0.02)	0.22^**^(0.02)	0.32^**^(0.03)
Age of child	0.09^**^(0.01)	−0.14^**^ (0.01)	0.07^**^(0.01)	0.06^** (^0.01)
Mother’s Education	−0.36^**^ (0.01)	−0.18^**^ (0.01)	−0.38^**^ (0.01)	−0.78^**^ (0.02)
Prenatal care: doctor	−0.14^**^ (0.01)	−0.11^**^ (0.01)	−0.15^**^ (0.01)	−0.49^**^ (0.02)
Sex of the child	0.09^**^ (0.01)	0.11^**^ (0.01)	0.09^**^ (0.01)	0.02 (0.02)
Birth order number	0.06^**^ (0.005)	0.01^*^ (0.006)	0.04^**^ (0.005)	0.22^**^ (0.007)
Number of antenatal visits during pregnancy	−0.05^**^ (0.015)	0.01 (0.017)	−0.05^**^ (0.015)	−0.47^**^ (0.023)
Constant	−0.65^**^ (0.09)	−0.76^**^ (0.10)	−0.70^**^ (0.09)	−1.11^**^ (0.14)
LR chi2	2943.19**	1854.37**	3598.06**	12532.21**
Observations	103787	101498	106313	110540

Notes: Standard errors are provided in parentheses.

The significance levels are marked as follows: * denotes 5% and ** denotes 1% level of significance (* *p* < 0.05, ^**^ *p* < 0.01)

**Table 4:** Regression results for problems with access to healthcare.

SDG Health Outcomes	(Dependent Variables)
	Problems with Access to Healthcare
Explanatory Variables	
Climatic Vulnerability	0.009^** (^0.00)
Woman’s Education	−0.13^**^ (0.00)
Woman’s age	−0.00^**^ (0.00)
Current marital status	0.02^**^(0.00)
Problem in getting a person to accompany the woman to a health facility	1.99^**^(0.00)
Problem in accessing transportation for the travel to a health facility	2.40^**^(0.00)
Constant	0.10^**^(0.00)
LR chi2	1.50e + 11**
Observations	447348

Notes: Standard errors are provided in parentheses.

The significance levels are marked as follows: * denotes 5% and ** denotes 1% level of significance (^*^ *p* < 0.05, ^**^ *p* < 0.01)

The coefficients for the climatic vulnerability variable indicate the log-odds of observing the associated health outcome in highly vulnerable districts as compared to the other districts. For example, in stunting (estimation 1, Table 3), the climatic vulnerability coefficient is 0.14, indicating a positive association, where individuals in highly vulnerable districts have 0.14 times higher log-odds of being stunted. Similarly, for wasting and being underweight, coefficients of 0.06 and 0.22 respectively, also indicate that there exists a positive association between higher climatic vulnerability and adverse outcomes on wasting and being underweight. The highest value for a positive coefficient is for non-institutional deliveries at 0.32, revealing a high likelihood of births occurring outside health-facilities in highly vulnerable districts as compared to other districts. Problems in accessing healthcare are also positively associated with climatic vulnerability, and are statistically significant, though the strength of the association is lower at a coefficient value of 0.009, indicating a small increase in the log-odds. All associations are statistically significant at 1% significance level (p values<0.01). The analysis also reveals that there is variation in the extent of the association of climatic vulnerability and health outcomes.

It is to be noted that these results hold after introducing other determinants of health outcomes in the estimation. The signs and significance levels obtained on other explanatory variables confirm to expectations in almost all the cases. For instance, the negative and significant association between mothers/women’s education and adverse health outcomes are well-established. As the level of education increases, the likelihood of an adverse health outcome reduces in all cases, with the coefficients being negative (and statistically significant) for stunting (−0.36), wasting (−0.18), underweight (−0.38), non-institutional deliveries (−0.78) and facing problems in accessing healthcare (−0.13). Similarly, receiving prenatal care from a doctor significantly reduces the log-odds of an individual being stunted, wasted, underweight and having non-institutional deliveries. Climatic vulnerability clearly plays a role in determining health outcomes, with high vulnerability levels being associated with more adverse outcomes.

A sensitivity analysis was conducted by using alternative groupings of vulnerability for each health outcome (dependent variable). Regressions were run using all 5 categories, and with alternative groupings across vulnerability categories. Very similar results are obtained for outcomes on stunting, wasting, underweight, and non-institutional deliveries. The signs of all explanatory variables remain the same (correctly signed) and significant with the outcome variables demonstrating an inverse relationship with climatic vulnerability, which intensifies with higher magnitudes of climatic vulnerability. For the access to health care outcome, the transportation availability variable was found to be significantly and highly correlated (chi square test) with the climatic vulnerability variable and the availability of a person to accompany the woman in an alternative grouping, which could be taken care of by dropping the transportation availability variable. The results from the alternative regressions confirm that our results are robust and reliable. (S7 Table, S8 Table and S9 Table present results from the sensitivity analysis).

**Fig 3:**
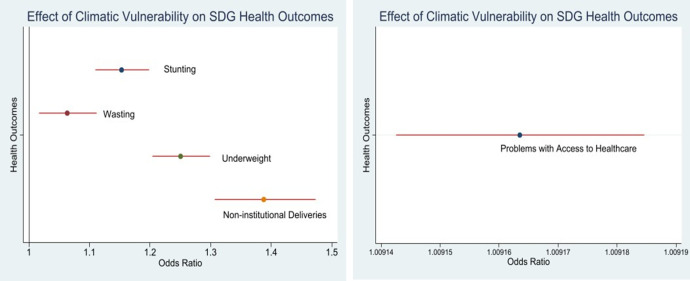
Odds ratios.

Fig 3 provides a graphical representation of the results, using the odds ratios from the estimations. The dots represent the odds ratios and the red lines show the corresponding 95% confidence intervals. The odds ratio for the outcome variables are greater than 1. This indicates that individuals living in highly vulnerable districts have a higher likelihood of experiencing adverse health outcomes as compared to those in districts that are less vulnerable to climate change. The chances of being stunted, wasted, or underweight are 1.15 (CI: 1.109–1.198), 1.06 (CI: 1.017–1.112) and 1.25 (CI: 1.203–1.299) times higher respectively, for districts that are highly vulnerable to climate change. In these highly vulnerable districts, the chances of women having non-institutional deliveries or facing problems in accessing health care is also 1.38 (CI: 1.307–1.473) and 1.009 (CI: 1.00914–1.00918) times higher, respectively.

The variation in odds ratios across health outcomes reveal that these are not equally sensitive to climatic vulnerabilities and that some outcomes are impacted more than others. The largest impact is observed on non-institutional deliveries, followed by being underweight and stunted. The distribution of wasting outcomes is more complex in the Indian context and finds higher prevalence across all the socioeconomic categories including amongst households in the higher wealth quintiles. In this regard, discerning a significant association with climatic vulnerability is of particular interest and suggests a potential role of the climatic factor in influencing the health outcomes.

## 4. Discussion

This study highlights the criticality of the role of climatic vulnerability in determining key health-related SDG targets in India. Our findings reveal that climate change has a significant and distinct association with poor health outcomes even after accounting for other well-established determinants for good health such as mothers’ education, birth orders of children, and access to health care facilities, in alignment with findings from major assessments [4,86–88].

The analysis reveals significant disparities in SDG outcomes across districts in India, in spite of progress being made in several of the determinants that are well-established as determinants of health outcomes. Districts which are highly vulnerable in terms of climate, underperform on health metrics including stunting, wasting, being underweight, having non-institutional deliveries and facing problems in accessing health care. Similar adverse findings for maternal and child health and nutrition attributable to climate change have been observed elsewhere [89–91] The study findings thus add value to the existing understanding on determinants of health outcomes in India, by providing substantive evidence at scale on how climate impacts act as an additional determinant. It innovatively brings together data from large and well-established datasets in India. The quantification of adverse impacts on health outcomes that can be attributed to climate change, establishes the extent to which climate risks can explain some of the persisting disparities in health outcomes. These risks need to be considered in health sector assessments health sector preparedness in line with the WHO call to action for protecting maternal and child health [92].

Cost-effective investment decisions need to consider the differentials in climate vulnerability across sub-national regions in health sector decision-making for effective implementation [93]. Tackling climatic vulnerability is essential for progressing on the SDG targets [94], and ignoring these can lead to delays and setbacks, most of which are avoidable. Effective climate resilient practices include communities in implementation and planning, empower local governance [95] and strengthen self-help groups [96] to enhance adaptive capacity. Since the current study uses data from a cross-sectional household survey it can provide inferences on the association between climate conditions and child health outcomes. Cohort studies can be designed specifically to overcome the limitations of such cross-section datasets, such as the presence of potentially unobservable factors that may be masked in a single large scale dataset. Further, in-depth studies are required to establish locally relevant and regionally disaggregated results, which could strengthen the evidence base on how local climatic conditions undermine SDG outcomes. Localized district level assessments can be particularly important for areas highly vulnerable to climatic extremes such as cyclones, droughts, floods and heatwaves. On the operational side, national level assessments can incorporate some of the climatic vulnerability related indicators.

## 5. Conclusion

The study provides quantitative evidence to establish the existence of a significant climate impact and its potential to disrupt sustainable SDG achievements. As such it establishes the unequivocal need for adaptation and for building a climate resilient healthcare system at sub-national levels to ensure that the benefits of investments made in achieving good health and well-being can be fully realized. Differentials in climate vulnerability need to be mainstreamed into plans and strategies for the health sector such as through the District Action Plans and the National Health Mission. State Action Plans on Climate Change, National Adaptation Plans and State Action Plans on Climate Change and Health are in the process of being drafted and revised by different states in India and the evidence provided in this study could be a timely input.

Tracking climate disaster impacts and integrating these with health outcomes will lead to better health system preparedness, including surveillance, flood and heat resilient health infrastructure, stocking up on medicines and essential supplies according to vulnerability profiles, and training of personnel to respond in a sustained manner, including paramedics and community health workers. Closer co-ordination between health departments and other departments such as state disaster management authorities and nodal agencies for implementation of climate change action plans can be facilitated through incorporation in the National Health Mission as well as the National Indicator Framework to track the progress on SDGs.

Similar studies for other LMICs would be useful in planning effective strategies for climate action to support development, including awareness creation and raising resources. Advancing good health is linked to advancing on several of the non-health SDGs, including poverty alleviation (SDG1), promoting inclusive societies (SDG16), settlements (SDG11), sustainable use of ecosystems (SDG15), learning opportunities (SDG4), and productive employment (SDG8). It is important therefore that health and non-health sector actors are made aware of the evidence on the impacts of climate change on health in order to eliminate disparities and progress on all the SDGs.

## Supporting information

S1 Fig Distribution of very high and highly vulnerable districts across India. (DOCX)

S1 Table Distribution of observations by level of climatic vulnerability. (DOCX)

S2 Table Description of dependent variables. (DOCX)

S3 Table Descriptive statistics on access to health care. (DOCX)

S4 Table Description of explanatory variables. (DOCX)

S5 Table VIF results on stunting, wasting, underweight, non-institutional deliveries. (DOCX)

S6 Table VIF results on problems with access to healthcare. (DOCX)

S7 Table Results on sensitivity analysis for stunting, wasting, underweight and non-institutional deliveries. (DOCX)

S8 Table Results on sensitivity analysis for problems in access to healthcare. (DOCX)

S9 Table Alternative results for regression on problems in access to healthcare. (DOCX)
