# Supplementary material for: Health SDGs are at risk from climate change: Evidence from India
Source: PLoS One. 2025 Nov 26;20(11):e0335529. doi: 10.1371/journal.pone.0335529 (PMC12654917; doi:10.1371/journal.pone.0335529)
Supplement: S1 Fig — (DOCX) [file pone.0335529.s001.docx]

**S1 Fig.** Distribution of Very High and Highly Vulnerable Districts across India

***
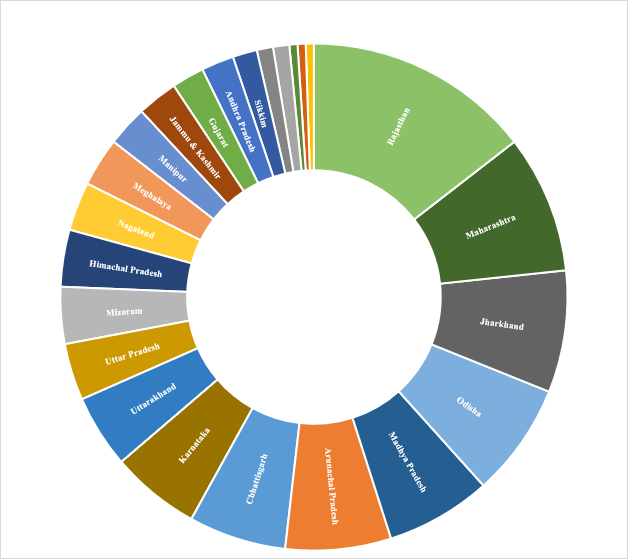
***

Note: Each segment in the figure above, represents the combined share of very highly and highly vulnerable districts in the state named, expressed as a proportion of the total number of such districts in India.
