# Supplementary material for: Health SDGs are at risk from climate change: Evidence from India
Source: PLoS One. 2025 Nov 26;20(11):e0335529. doi: 10.1371/journal.pone.0335529 (PMC12654917; doi:10.1371/journal.pone.0335529)
Supplement: S1 Table — (DOCX) [file pone.0335529.s002.docx]

**S****1 Table.** Distribution of observations by level of climatic vulnerability

| Climatic Vulnerability | Percentage share |
| --- | --- |
| Very High | 5 |
| High | 32 |
| Medium | 37 |
| Low | 20 |
| Very low | 6 |
