# Supplementary material for: Health SDGs are at risk from climate change: Evidence from India
Source: PLoS One. 2025 Nov 26;20(11):e0335529. doi: 10.1371/journal.pone.0335529 (PMC12654917; doi:10.1371/journal.pone.0335529)
Supplement: S2 Table — (DOCX) [file pone.0335529.s003.docx]

**S2 Table.** Description of Dependent Variables

| Dependent Variables | Description |
| --- | --- |
| Stunting | Height-for-age is a measure of linear growth retardation and cumulative growth deficits. Children whose height-for-age Z-score is below minus two standard deviations (-2 SD) from the median of the reference population are considered short for their age (stunted), or chronically undernourished (IIPS 2021). |
| Wasting | Weight-for-height index measures body mass in relation to body height or length and describes current nutritional status. Children whose Z-score is below minus two standard deviations (-2 SD) from the median of the reference population are considered thin (wasted), or acutely undernourished (IIPS 2021). |
| Underweight | Weight-for-age is a composite index of height-for age and weight-for-height. It covers acute and chronic undernutrition. Children whose weight-for-age Z-score is below minus two standard deviations (-2 SD) from the median of the reference population are classified as underweight (IIPS 2021). |
| Institutional deliveries | Institutional delivery refers to giving birth with the help of trained health professionals, under safe and sterile procedures. NFHS provides a percent distribution of all live births to women aged 15-49 in the five years preceding the survey by place of births. (IIPS 2021) |
| Access to Healthcare | It is defined as the extent to which women in the age group of 15-49 encounter challenges in obtaining medical treatment for themselves when they are sick (IIPS 2021). |
