# Supplementary material for: Health SDGs are at risk from climate change: Evidence from India
Source: PLoS One. 2025 Nov 26;20(11):e0335529. doi: 10.1371/journal.pone.0335529 (PMC12654917; doi:10.1371/journal.pone.0335529)
Supplement: S3 Table — (DOCX) [file pone.0335529.s004.docx]

**S3 Table.** Descriptive statistics on *Access to Health Care*

| Health Outcome | Variables | Level | Percentage Distribution (%) |
| --- | --- | --- | --- |
| Access to Health Care | Distance to health care | Big Problem | 29.08 |
|  |  | Small Problem | 35.78 |
|  |  | No Problem | 35.14 |
|  | Non-availability of health care professional | Big Problem | 44.15 |
|  |  | Small Problem | 26.40 |
|  |  | No Problem | 29.45 |
|  | Non-availability of female health care professional | Big Problem | 34.46 |
|  |  | Small Problem | 31.16 |
|  |  | No Problem | 34.38 |
|  | Non-availability of drugs | Big Problem | 45.57 |
|  |  | Small Problem | 24.81 |
|  |  | No Problem | 29.62 |
