# Supplementary material for: Health SDGs are at risk from climate change: Evidence from India
Source: PLoS One. 2025 Nov 26;20(11):e0335529. doi: 10.1371/journal.pone.0335529 (PMC12654917; doi:10.1371/journal.pone.0335529)
Supplement: S4 Table — (DOCX) [file pone.0335529.s005.docx]

**S4 Table.** Description of explanatory variables

| Explanatory Variables | Description |
| --- | --- |
| Age of Children: | The age of children under five years is provided in both completed months and completed years in kids file. |
| Sex of Child: | The sex of the child is recorded as a categorical variable coded as male or female. |
| Age of women: | Age of women (15-49 years) is recorded as a numeric variable indicating their completed age in years at the time of the survey. |
| Mother's Education/Woman's Education: | Information on education for mothers is recorded in terms of the highest level of education completed and the total number of completed years of schooling. This information is provided in the children's file for mothers and in the women's individual file for women of reproductive age. |
| Marital Status: | Marital status for women is categorized as a discrete variable, typically with values such as never married, currently married, widowed, divorced, or separated. |
| Number of Antenatal Visits During Pregnancy: | The total number of antenatal visits during pregnancy is provided in the kids file for the children born in last five years preceding the survey as a numeric variable, reflecting how many times mother sought healthcare for ANC during her pregnancy. |
| Mother Received Prenatal Care from Doctor: | Information on prenatal care during pregnancy during last five preceding the survey has been given in kids file and recorded as a binary variable, reflecting whether the mother received prenatal care from a doctor during her pregnancy. The binary coding (1 = Yes, 0 = No) is used to analyze the access to and utilization of professional healthcare services during pregnancy. |
| Problem in getting a person to accompany the woman to a health facility | It is defined as the extent to which women in the age group of 15-49 encounter challenges in finding someone to accompany them when seeking healthcare services.  The responses for this variable is given in categorical form, which includes 'Big problem,' 'Small problem,' and 'No problem.' We have recoded these variables into a binary form by combining 'Small problem' and 'No problem' into one category, with an assigned value of '0,' and 'Big problem' with an assigned value of '1’. |
| Problem in accessing transportation for the travel to a health facility | It is defined as the extent to which women in the age group of 15-49 encounter challenges in accessing a mode of transportation when attempting to seek healthcare. The responses for this variable are given in categorical form, which include 'Big problem,' 'Small problem,' and 'No problem.' We have recoded these variables into a binary form by combining 'Small problem' and 'No problem' into one category, with an assigned value of '0,' and 'Big problem' with an assigned value of '1’. |
