# Supplementary material for: Health SDGs are at risk from climate change: Evidence from India
Source: PLoS One. 2025 Nov 26;20(11):e0335529. doi: 10.1371/journal.pone.0335529 (PMC12654917; doi:10.1371/journal.pone.0335529)
Supplement: S5 Table — (DOCX) [file pone.0335529.s006.docx]

**S5. Table** VIF results on Stunting, Wasting, Underweight and Non-institutional deliveries

|  | VIF | | | |
| --- | --- | --- | --- | --- |
| SDG health outcome  (dependent variable) | Stunting | Wasting | Underweight | Non institutional deliveries |
| Explanatory variables |  |  |  |  |
| Climatic Vulnerability | 1.7 | 1.71 | 1.7 | 1.69 |
| Age of child | 1.03 | 1.03 | 1.03 | 1.03 |
| Mother's education | 1.27 | 1.28 | 1.27 | 1.27 |
| Prenatal care: doctor | 1.24 | 1.24 | 1.24 | 1.24 |
| Sex of the child | 1 | 1 | 1 | 1 |
| Birth order number | 1.24 | 1.24 | 1.24 | 1.24 |
| Number of antenatal visits during pregnancy | 1.23 | 1.24 | 1.24 | 1.23 |
