# Supplementary material for: Health SDGs are at risk from climate change: Evidence from India
Source: PLoS One. 2025 Nov 26;20(11):e0335529. doi: 10.1371/journal.pone.0335529 (PMC12654917; doi:10.1371/journal.pone.0335529)
Supplement: S6 Table — (DOCX) [file pone.0335529.s007.docx]

**S6 Table.** VIF results on Problems with Access to Healthcare

|  | VIF |
| --- | --- |
| SDG health outcome (dependent variable) | Problems with Access to Healthcare |
| Explanatory variables |  |
| Climatic Vulnerability | 1.66 |
| Woman’s Education | 1.37 |
| Woman’s age | 1.62 |
| Current marital status | 1.34 |
| Problem in getting a person to accompany the woman to a health facility | 1.38 |
| Problem in accessing transportation for the travel to a health facility | 1.38 |
