# Supplementary material for: Health SDGs are at risk from climate change: Evidence from India
Source: PLoS One. 2025 Nov 26;20(11):e0335529. doi: 10.1371/journal.pone.0335529 (PMC12654917; doi:10.1371/journal.pone.0335529)
Supplement: S7 Table — (DOCX) [file pone.0335529.s008.docx]

**S7 Table.** Results on sensitivity analysis for Stunting, Wasting, Underweight and Non-institutional deliveries (regression after regrouping the climatic vulnerability variable).

|  | (1) | (2) | (3) | (4) |
| --- | --- | --- | --- | --- |
| SDG health outcome (dependent variable) | Stunting | Wasting | Underweight | Non-institutional deliveries |
| Explanatory variables |  |  |  |  |
| Climatic vulnerability | 0.123^**^ | 0.102^**^ | 0.153^**^ | 0.125^**^ |
|  | (0.019) | (0.022) | (0.019) | (0.029) |
|  |  |  |  |  |
| Age of child | 0.093^**^ | -0.140^**^ | 0.071^**^ | 0.059^**^ |
|  | (0.005) | (0.006) | (0.005) | (0.007) |
|  |  |  |  |  |
| Mothers Education | -0.363^**^ | -0.183^**^ | -0.392^**^ | -0.794^**^ |
|  | (0.015) | (0.018) | (0.015) | (0.022) |
|  |  |  |  |  |
| Prenatal care from doctor | -0.142^**^ | -0.117^**^ | -0.159^**^ | -0.495^**^ |
|  | (0.015) | (0.017) | (0.015) | (0.022) |
|  |  |  |  |  |
| Sex of child | 0.092^**^ | 0.112^**^ | 0.098^**^ | 0.024 |
|  | (0.013) | (0.016) | (0.013) | (0.020) |
|  |  |  |  |  |
| birth order number | 0.062^**^ | 0.013^*^ | 0.047^**^ | 0.221^**^ |
|  | (0.005) | (0.006) | (0.005) | (0.007) |
|  |  |  |  |  |
| ANC checkups | -0.056^**^ | 0.018 | -0.060^**^ | -0.478^**^ |
|  | (0.015) | (0.017) | (0.015) | (0.023) |
|  |  |  |  |  |
| LR chi2 | 2934.09** | 1868.04** | 3534.30** | 3534.30** |

Standard errors in parentheses

^*^ *p* < 0.05, ^**^ *p* < 0.01
