# Supplementary material for: Health SDGs are at risk from climate change: Evidence from India
Source: PLoS One. 2025 Nov 26;20(11):e0335529. doi: 10.1371/journal.pone.0335529 (PMC12654917; doi:10.1371/journal.pone.0335529)
Supplement: S8 Table — (DOCX) [file pone.0335529.s009.docx]

**S8 Table.** Results on sensitivity analysis for ‘Problems in access to healthcare’ (regression after regrouping climatic vulnerability variable and dropping the transport variable).

|  | (1) |
| --- | --- |
| SDG health outcome (dependent variable) | Problems in access to healthcare |
| Explanatory variables |  |
| Climatic vulnerability | 0.006^**^ |
|  | (0.000) |
|  |  |
| Educational Attainment | -0.211^**^ |
|  | (0.000) |
|  |  |
| Age of women | -0.004^**^ |
|  | (0.000) |
|  |  |
| Marital status | -0.004^**^ |
|  | (0.000) |
|  |  |
| Nature of the problem in getting a person to accompany the woman to a health facility | 2.836^**^ |
|  | (0.000) |
|  |  |
| LR chi2 | 1.02e+11** |

Standard errors in parentheses

^*^ *p* < 0.05, ^**^ *p* < 0.01
